# Immunostimulatory gene therapy targeting CD40, 4-1BB and IL-2R activates DCs and stimulates antigen-specific T-cell and NK-cell responses in melanoma models

**DOI:** 10.1186/s12967-023-04374-2

**Published:** 2023-07-27

**Authors:** Jessica Wenthe, Emma Eriksson, Ann-Charlotte Hellström, Rafael Moreno, Gustav Ullenhag, Ramon Alemany, Tanja Lövgren, Angelica Loskog

**Affiliations:** 1 Department of Immunology, Genetics and Pathology, Science for Life Laboratory, Rudbeck Laboratory, Uppsala University, Dag Hammarskjöldsväg 20, 751 85 Uppsala, Sweden; 2 Lokon Pharma AB, Uppsala, Sweden; 3 IDIBELL-Institute Català d’Oncologia, L’Hospitalet de Llobregat, Barcelona, Spain; 4 Department of Oncology, Uppsala University Hospital, Uppsala, Sweden

**Keywords:** Oncolytic adenovirus, Immunotherapy, Dendritic cells, T cells, IL-2, CD40L, 4-1BBL

## Abstract

**Background:**

The activation of dendritic cells (DCs) is pivotal for generating antigen-specific T-cell responses to eradicate tumor cells. Hence, immunotherapies targeting this interplay are especially intriguing. Moreover, it is of interest to modulate the tumor microenvironment (TME), as this harsh milieu often impairs adaptive immune responses. Oncolytic viral therapy presents an opportunity to overcome the immunosuppression in tumors by destroying tumor cells and thereby releasing antigens and immunostimulatory factors. These effects can be further amplified by the introduction of transgenes expressed by the virus.

**Methods:**

Lokon oncolytic adenoviruses (LOAd) belong to a platform of chimeric serotype Ad5/35 viruses that have their replication restricted to tumor cells, but the expression of transgenes is permitted in all infected cells. LOAd732 is a novel oncolytic adenovirus that expresses three essential immunostimulatory transgenes: trimerized membrane-bound CD40L, 4-1BBL and IL-2. Transgene expression was determined with flow cytometry and ELISA and the oncolytic function was evaluated with viability assays and xenograft models. The activation profiles of DCs were investigated in co-cultures with tumor cells or in an autologous antigen-specific T cell model by flow cytometry and multiplex proteomic analysis. Statistical differences were analyzed with Kruskal–Wallis test followed by Dunn’s multiple comparison test.

**Results:**

All three transgenes were expressed in infected melanoma cells and DCs and transgene expression did not impair the oncolytic activity in tumor cells. DCs were matured post LOAd732 infection and expressed a multitude of co-stimulatory molecules and pro-inflammatory cytokines crucial for T-cell responses. Furthermore, these DCs were capable of expanding and stimulating antigen-specific T cells in addition to natural killer (NK) cells. Strikingly, the addition of immunosuppressive cytokines TGF-β1 and IL-10 did not affect the ability of LOAd732-matured DCs to expand antigen-specific T cells and these cells retained an enhanced activation profile.

**Conclusions:**

LOAd732 is a novel immunostimulatory gene therapy based on an oncolytic adenovirus that expresses three transgenes, which are essential for mediating an anti-tumor immune response by activating DCs and stimulating T and NK cells even under imunosuppressive conditions commonly present in the TME. These qualities make LOAd732 an appealing new immunotherapy approach.

**Supplementary Information:**

The online version contains supplementary material available at 10.1186/s12967-023-04374-2.

## Background

The aim of cancer immunotherapy is to activate or re-activate an anti-tumor immune response. To achieve an antigen-specific immunity, it is crucial to have functional dendritic cells (DCs) that are capable of presenting tumor antigens to T cells (signal 1) while providing co-stimulatory signals (signal 2) and pro-inflammatory cytokines (signal 3). All three signals are required to allow for efficient expansion of antigen-specific T cells and eradication of tumor cells [1]. However, the microenvironment of tumors is often immunosuppressive, and many cells or factors required for establishing an immune response are inhibited or missing. Oncolytic viruses can modulate the tumor microenvironment (TME) by infecting and killing tumor cells, which then release tumor-associated antigens and immunostimulatory factors. Moreover, oncolytic viruses can be further engineered to express essential immunostimulatory transgenes [2].

Lokon oncolytic adenoviruses (LOAd) are serotype Ad5/35 viruses and their replication is restricted to tumor cells harboring a dysregulated retinoblastoma pathway due to a deletion in the adenoviral gene E1A (Δ24). In contrast, the transgene expression is controlled by a CMV promoter, which enables transgene expression not only in tumor cells, but in all infected cells in the injected TME, such as endothelial, stromal and immune cells. We have previously investigated LOAd703 (delolimogene mupadenorepvec), expressing trimerized membrane-bound (TMZ)-CD40L and 4-1BBL, extensively in pancreatic cancer, multiple myeloma, and in combination with chimeric antigen receptor T cells in B-cell lymphoma and with checkpoint blockade in melanoma [3–7]. Currently, LOAd703 is assessed in various clinical trials for the treatment of malignant melanoma, pancreatic, ovarian, colorectal and biliary cancer (NCT02705196, NCT03225989, NCT04123470).

Herein, we present the first evaluation of the novel LOAd virus named LOAd732. Like LOAd703, LOAd732 expresses TMZ-CD40L and 4-1BBL, but it is additionally armed with a third essential factor for driving T-cell responses: IL-2. Hence, the aim of this paper is to validate the functionality of the new construct expressing three transgenes and if the addition of IL-2 will further spark lymphocyte activation. Signaling of CD40L/CD40 in DCs is known to be a potent DC activator, leading to mature DCs expressing co-stimulatory molecules and cytokines, which then are able to initiate an antigen-specific T-cell response [8]. T-cell stimulation is supported by 4-1BBL/4-1BB signaling, leading to enhanced effector function and survival. The expansion of activated T cells is further potentiated by IL-2. Both 4-1BBL and IL-2 are also effective natural killer (NK) cell stimulators [9, 10]. The proposed impact on T and NK cells by LOAd732 was evaluated in this study in human in vitro models and under suppressive conditions mimicking the TME by exposure to TGF-β1 and IL-10, which are two immunosuppressive factors overexpressed in malignant melanoma [11, 12].

## Methods

### Cell culture

Human melanoma cells Mel526 (RRID:CVCL_8051) were provided by Prof. Magnus Essand (Uppsala University, Uppsala, Sweden) and Mel624 cells (RRID:CVCL_8054) were a kind gift from Prof. Rolf Kiessling (Karolinska Institute, Stockholm, Sweden). Melanoma cells were cultured in RPMI-1640 supplemented with penicillin (100 U/mL)/streptomycin (100 µg/mL) (PeSt) and 10% fetal bovine serum (FBS). Human monocyte-derived DCs and co-cultures with autologous T cells were cultured in RPMI-1640 supplemented with PeSt, 10mM HEPES, 0.02mM 2-mercaptoethanol and 10% FBS. Cells were cultured at 37 °C, 5% CO_2_. Media and supplements were purchased from Thermo Fisher Scientific (Waltham, MA, USA).

### Virus construction and infection

Lokon oncolytic adenoviruses (LOAd) were provided by Lokon Pharma, AB, Uppsala, Sweden. The method to generate LOAd viruses have been described previously [13]. For LOAd732, a plasmid containing the transgene cassette was obtained from GenScript (Piscataway, NJ, USA) and then cloned into the LOAd viruses. LOAd viruses are oncolytic serotype 5/35 adenoviruses, which express immunostimulatory transgenes under the control of a CMV promoter. LOAd703 [3] and LOAd732 both encode for trimerized membrane-bound CD40L (TMZ-CD40L) and 4-1BBL, but LOAd732 additionally encodes for IL-2. As a control, an oncolytic LOAd virus lacking the transgene cassette was used (LOAd(-)). The virus concentration is presented as fluorescent forming units (FFU)/ml and represents viable infectious viruses. For infection, cells were washed and pelleted in serum-free medium before the respective virus (10–100 FFU/cell) was added and incubated for two hours at 37 °C, 5% CO_2_, followed by addition of complete growth medium.

### Transgene expression analysis

Melanoma cells were infected with 10 FFU/cell of the respective virus and cultured for 48h before supernatants and cells were harvested for transgene expression analysis. IL-2 levels in supernatants were determined with human IL-2 ELISA development kit from Mabtech (Nacka Strand, Sweden). Cell surface expression of CD40L and 4-1BBL was analyzed with flow cytometry. For this, cells were stained with fluorescent-labeled antibodies (BioLegend, San Diego, CA, USA) against CD40L (RRID:AB_2562721) and 4-1BBL (RRID:AB_314883) and respective isotype control antibodies.

### Flow cytometry

Cells were washed in PBS supplemented with 3mM EDTA (Thermo Fisher) and 0.5% bovine serum albumin (Sigma-Aldrich) and stained with fluorescent-labeled antibodies according to the specific experiment. After staining, cells were washed with fixation buffer (PBS with 1% formaldehyde and 3mM EDTA) and analyzed with BD FACS Canto 2 (BD Biosciences) and FlowJo software (FlowJo LLC).

### Cell viability assay

Melanoma cells were infected with 100 FFU/cell of the respective virus, plated in quadruplicates and analyzed for viability 48h post infection with CellTiter 96 AQueous One Solution MTS reagent (Promega, Fitchburg, WI, USA).

### Xenograft model

Animal experiments were approved by the local animal ethical review board in Uppsala, Sweden (DNr 5.8.18-08932/2019). Melanoma cells were mixed 1:1 with Corning Matrigel matrix (Corning, NY, USA) and injected subcutaneously in female immunodeficient BALB/c nude mice (5 × 10^6^ cells/mouse; 5 mice per group). Treatments with LOAd732 or LOAd(-) (1 × 10^9^ FFU/mouse), or saline as control, were initiated when tumors were detectable in at least 50% of mice (Mel526: day 7, Mel624: day 17/18) and given at the site of tumor injection/intratumorally two times per week for 3 weeks. Tumor growth was monitored by measuring the ellipsoid tumor volume and mice with tumors over 1000 mm^3^ were sacrificed.

### Dendritic cell maturation analysis

Peripheral blood mononuclear cells (PBMCs) were isolated from healthy blood donor buffy coats acquired from the blood bank at Uppsala University hospital. CD14 + cells were purified with CD14 MicroBeads (RRID:AB_2665482) (Miltenyi Biotec, Bergisch Gladbach, Germany) and cultured for 6 days with 150 ng/mL of GM-CSF and 50 ng/mL of IL-4 (Peprotech, Rocky Hill, NJ, USA). Immature DCs were infected with the respective virus (50 FFU/cell) or left untreated. DCs were cultured alone or co-cultured with Mel526 cells infected with the respective virus (DC:Mel526 ratio = 2:1). Prior co-culture, LOAd-infected Mel526 cells were washed with serum-free medium to remove presence of the virus in supernatants. 48h post infection, cell culture supernatants were harvested and analyzed for the expression of cytokines and chemokines with U-PLEX multiplex analysis from Meso Scale Diagnostics (Rockville, MD, USA) according to manufacturer’s instructions. Cells were harvested and stained with the following fluorescent-labeled antibodies (BioLegend) and respective isotype controls: anti-CD40L (RRID:AB_2562721), anti-4-1BBL ((RRID:AB_314883), anti-CD70 (clone: 113-16), anti-CD80 (RRID:AB_2076147), anti-CD83 (RRID:AB_314516), anti-CD86 (RRID:AB_11204252), anti-HLA-ABC (RRID:AB_314873), anti-HLA-DR (RRID:AB_314688), anti-CCR7 (RRID:AB_11203894), anti-ICAM-1 (RRID:AB_2121926), anti-PD-L1 (RRID:AB_940358) and anti-CD1a (RRID:AB_2744317, BD Biosciences).

### Antigen-specific T-cell expansion analysis (CMV assay)

PBMCs were isolated from cytomegalovirus (CMV) seropositive healthy blood donor buffy coats acquired from the blood bank at Uppsala University hospital. PBMCs were screened with flow cytometry for HLA-A2 expression (RRID:AB_1659245) and presence of CD8 + T cells specific for the CMV peptide pp65 (iTAg Tetramer/PE – HLA-A*02:01 CMV pp65 (NLVPMVATV), MBL international, Woburn, MA, USA). CD14 + cells and T cells were purified from CMV tetramer positive donors with CD14 MicroBeads and Pan T Cell Isolation Kit, respectively (Miltenyi Biotec). CD14 + cells were cultured for 2 days with 150 ng/mL of GM-CSF and 50 ng/mL of IL-4 (Peprotech) and then either left untreated, stimulated with 40 ng/mL TNF-α and 30 µg/mL Poly(I:C) (Sigma-Aldrich, St. Louis, MO, USA), or infected with the respective LOAd viruses (40–50 FFU/cell). 24 h later, DCs were harvested, washed in PBS and pulsed with 0.01 µg/mL of CMV pp65 NLVPMVATV peptide (GenScript, Piscataway, NJ, USA) and 1 µg/mL of β2-microglobulin for one hour. DCs were washed in complete medium and co-cultured with autologous T cells in a 1:10 ratio for 11 days. For experiments under suppressive conditions, 2.5 ng/mL IL-10 (BioLegend) and 10 ng/mL TGF-β1 (Sigma-Aldrich) were added to the co-cultures [14]. At day 11, supernatants were taken for multiplex analysis with Olink Target 96 Immuno-Oncology (Olink Proteomics, Uppsala, Sweden) and cells were harvested for flow cytometry analysis of expanded cells. Cells were stained with Zombie NIR™ viability dye (BioLegend), iTAg Tetramer/PE—HLA-A*02:01 CMV pp65, anti-CD3 (RRID:AB_893298), anti-CD8 (RRID:AB_314124), anti-PD-1 (RRID:AB_2159324), anti-TIM-3 (RRID:AB_11218598), anti-LAG-3 (RRID:AB_2728373), anti-CD107a (RRID:AB_2562648), anti-CCR7 (RRID:AB_11203894), anti-CD45RA (RRID:AB_2561947), anti-CD16 (RRID:AB_314206), anti-CD56 (RRID:AB_11218798) and respective isotype control antibodies if applicable (all BioLegend). The following antibodies were purchased from BD Biosciences: anti-CD3 (RRID:AB_2783791), anti-CD8 (RRID:AB_2868803), anti-CD4 (RRID:AB_1645732), anti-CD25 (RRID:AB_2783790), anti-CD127 (RRID:AB_2033938), anti-CD69 (RRID:AB_400353), anti-CD16 (RRID:AB_2868628), anti-CD56 (RRID:AB_2868831).

### Statistics

Statistical analysis was performed with GraphPad Prism 9 (La Jolla, CA, USA). Kruskal–Wallis test followed by Dunn’s multiple comparison test was used to determine statistically differences between treatment groups and control.

## Results

### LOAd732 infection induced the expression of three transgenes in melanoma cell lines

**Fig. 1:**
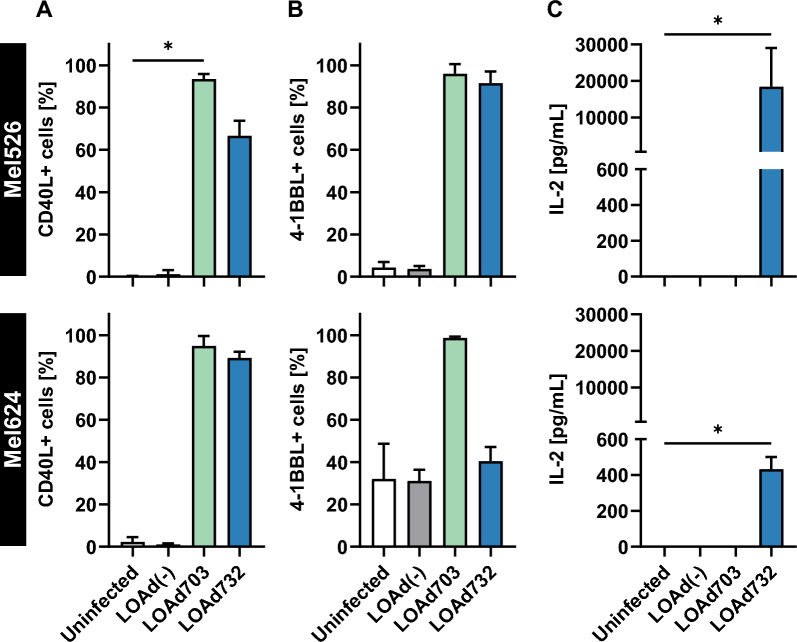
Transgene expression in melanoma cell lines. Melanoma cells Mel526 and Mel624 were infected with LOAd(-), LOAd703 or LOAd732, or left uninfected. 48 h later, cell culture supernatants and cells were harvested for transgene expression analysis. Expression of CD40L (**A**) and 4-1BBL (**B**) on the cells was determined with flow cytometry and is shown as percentage positive cells, whereas IL-2 concentrations (**C**) in the culture supernatants were measured with ELISA. The bar graphs show the mean ± SD of three biological repeats. Statistical differences between LOAd-infected and uninfected cells were determined with Kruskal–Wallis test followed by Dunn’s multiple comparison test (*p < 0.05)

To test the functionality of the new virus LOAd732, melanoma cell lines Mel526 and Mel624 were analyzed for the expression of the transgenes TMZ-CD40L, 4-1BBL and IL-2 post LOAd infection. Of note, both cell lines displayed some 4-1BBL expression at baseline. Over 90% of Mel526 and Mel624 cells expressed CD40L after LOAd703 infection and expression levels were slightly lower after LOAd732 infection (67% and 89% CD40L +) (Fig. 1A). Likewise, 4-1BBL expression was induced in over 95% in both cell lines after LOAd703 infection. However, LOAd732 infection only resulted in high 4-1BBL expression in Mel526 cells (92%), but not in Mel624 cells (40%) (Fig. 1B). IL-2 was detected in supernatants of both LOAd732-infected cell lines with higher concentrations in supernatants from Mel526 (~ 18,000 pg/mL) compared to Mel624 (~ 400 pg/mL) (Fig. 1C). All in all, the new LOAd732 construct is functional and can lead to the expression of three transgenes simultaneously.

### LOAd732 infection resulted in the killing of melanoma cell lines both in vitro and in vivo

**Fig. 2:**
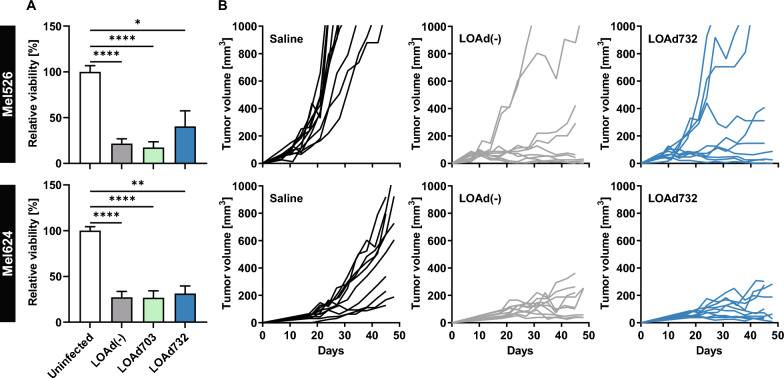
Oncolysis in vitro and in vivo. In A, melanoma cells Mel526 and Mel624 were infected with LOAd(-), LOAd703 or LOAd732, or left uninfected. 48 h later, the relative viability of the tumor cells was assessed with CellTiter 96 AQueous One Solution MTS reagent. The bar graphs show the pooled data of three biological repeats and the mean ± SD of technical quadruplicates. Statistical differences between LOAd-infected and uninfected cells were determined with Kruskal–Wallis test followed by Dunn’s multiple comparison test (*p < 0.05, **p < 0.01, ****p < 0.0001) (**A**). In B, Mel526 or Mel624 cells were injected subcutaneously in female immunodeficient BALB/c nude mice. Treatments with saline control, LOAd(-) or LOAd732 were initiated when tumors were detectable in at least 50% of mice (Mel526: day 7, Mel624: day 17/18) and given intratumorally two times per week for 3 weeks. Tumor growth was monitored and mice with tumors over 1000 mm^3^ were sacrificed. The graphs show the individual tumor growth curve for each mouse and the pooled data of two experimental repeats (10 mice in total per group) (**B**)

Next, the oncolytic function was confirmed in vitro as well as in vivo. LOAd732 infection resulted in the killing of Mel624 cells to the same degree as LOAd(-) and LOAd703 infection in vitro, but Mel526 cells showed a slightly higher viability post LOAd732 infection (Fig. 2A). The same cell lines were used to evaluate the oncolytic function of LOAd732 in xenograft models in vivo. As the immunostimulatory transgenes do not have effect in immunodeficient mice, only the LOAd732 was evaluated compared to LOAd(-) and saline control. LOAd703 has been previously analyzed in different xenograft models, where it showed a comparable effect to LOAd(-) [3, 5, 6]. Mel526 tumors grew more rapidly and not all tumors could be controlled by LOAd treatment and the effect seemed slightly worse for LOAd732 compared to the control virus LOAd(-), similarly to the in vitro data. In contrast, Mel624 tumors grew more slowly and could be controlled to the same extent by both LOAd(-) and LOAd732 (Fig. 2B). Taken together, the LOAd732 virus could control tumor growth in xenograft models of melanoma by its oncolytic capacity alone.

### DCs showed an activated phenotype profile post LOAd703/LOAd732 infection

**Fig. 3:**
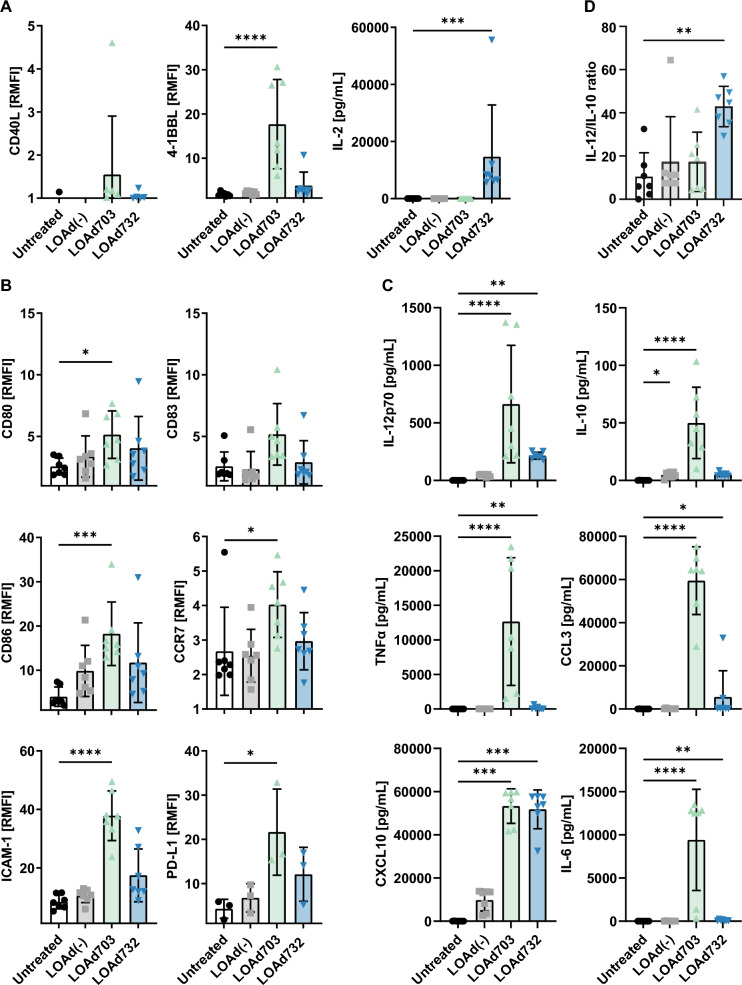
Activation of dendritic cells by LOAd viruses. Monocytes were isolated from peripheral blood mononuclear cells and cultured with GM-CSF/IL-4 to induce immature dendritic cells (DCs). DCs were infected with LOAd(-), LOAd703 or LOAd732, or left untreated. 48 h post infection, cell culture supernatants and cells were harvested and analyzed for DC maturation with flow cytometry and multiplex analysis. Flow cytometry results in A and B are displayed as relative mean fluorescence intensity (RMFI) compared to matched isotype control antibodies. DCs were gated on CD1a expression and the MFI of the respective marker was determined for CD1a + cells. In A, the transgene expression in DCs is shown (**A**) and B displays DC activation markers (**B**). In C, cytokine and chemokine levels in the culture supernatants are shown in pg/mL (**C**). D displays the ratio of IL-12 to IL-10 in culture supernatants (**D**). Bar graphs display the mean ± SD of seven biological repeats. Statistical differences between LOAd-infected and untreated cells were determined with Kruskal–Wallis test followed by Dunn’s multiple comparison test (*p < 0.05, **p < 0.01, ***p < 0.001, ****p < 0.0001)

The immunostimulatory effects of the virus and the transgenes can best be evaluated in human in vitro models since LOAd viruses infect cells via the human receptor CD46, which has no homologue in mice, and because of the limited cross reaction of the human transgenes with the respective mouse targets. We have previously shown that LOAd703, expressing TMZ-CD40L and 4-1BBL, is a potent DC stimulator [3]. Given this fact and the central role of DCs in generating anti-tumor immune responses, we aimed to evaluate if the new virus LOAd732, which additionally expresses IL-2, could still activate immature DCs. For this, monocyte-derived human DCs were infected with the LOAd viruses and analyzed for the expression of the transgenes and activation markers by flow cytometry. All three transgenes were expressed in LOAd732-infected DCs, but CD40L and 4-1BBL expression was lower than in LOAd703-infected DCs (Fig. 3A). Likewise, expression of co-stimulatory molecules CD80, CD83, CD86, homing molecules CCR7 and ICAM-1, and PD-L1 were highest in LOAd703-infected DCs, but also slightly upregulated in LOAd732-infected DCs compared to untreated cells (Fig. 3B). The expression of CD70, HLA-ABC, HLA-DR is shown in Additional file 1: Figure S1A. CD70 was highly upregulated on LOAd703-infected DCs and to a lesser extent on LOAd732-infected DCs. MHC class I and II molecules tended to be generally increased by LOAd virus infection. Overall, the same pattern with high upregulation with LOAd703 and LOAd732 to a lesser extent was observed with the secreted cytokines/chemokines (IL-12p70, IL-10, TNFα, CCL3, IL-6, IL-15, IL-21, IFNα2a, IFNγ) analyzed in the supernatants (Fig. 3C and Additional file 1: Figure S1A). CXCL10 expression was induced highly and to the same extent in both LOAd703- and LOAd732-infected DCs (Fig. 3C). IL-12 and IL-10 have opposing roles on DC activation and drive a Th1 or Th2 response, respectively [15]. Hence, a higher IL-12 to IL-10 ratio, which was observed with LOAd732 (Fig. 3D), is considered advantageous for stimulating antigen-specific cytotoxic T cells.

### Enhanced DC activation in co-cultures with LOAd732-infected melanoma cells

**Fig. 4:**
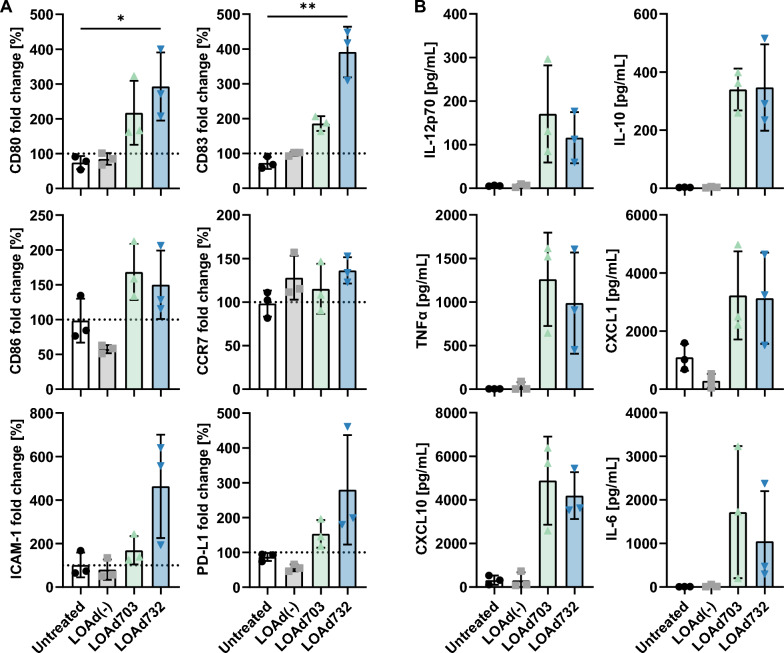
Activation of dendritic cells co-cultured with LOAd-infected melanoma cells. Monocytes were isolated from peripheral blood mononuclear cells and cultured with GM-CSF/IL-4 to induce immature dendritic cells (DCs). DCs were co-cultured with Mel526 cells that were either infected with LOAd(-), LOAd703 or LOAd732, or left untreated (DC:Mel526 ratio = 2:1). As control condition, DCs were infected directly with the respective LOAd virus or left untreated and cultured alone. Cells were cultured for 48 h before cell culture supernatants and cells were harvested and analyzed for DC maturation with flow cytometry and multiplex analysis. For flow cytometry analysis, DCs were gated based on CD1a expression and the mean fluorescence intensity of the respective marker was determined for CD1a + cells based on matched isotype control antibodies. The expression of activation markers in A is displayed as percentage fold change over control condition (DCs cultured alone) (**A**). In B, cytokine and chemokine levels in the culture supernatants of the co-cultures are shown in pg/mL (**B**). Bar graphs display the mean ± SD of three biological repeats. Statistical differences between LOAd-infected and untreated cells were determined with Kruskal–Wallis test followed by Dunn’s multiple comparison test (*p < 0.05, **p < 0.01)

In the previous experiment, the DCs were directly infected with the virus, but to better mimic the situation in the patient and TME, we wanted to explore the activation of DCs in co-culture with LOAd-infected tumor cells (Mel526). Figure 4A displays the expression of activation markers as fold change to the levels observed in DCs that were infected directly and cultured alone. Co-culture with Mel526 cells did not inhibit DC activation upon LOAd703/LOAd732 infection as there was no reduction in the investigated markers compared to the DCs infected and cultured alone. In contrast, the activation markers were further upregulated with both LOAd703- and LOAd732-infected tumor cells. Intriguingly, co-culture with LOAd732-infected tumor cells resulted in the highest fold change increase of activation markers and in the secretion of comparable levels of cytokines and chemokines between LOAd703 and LOAd732 (Fig. 4B and Additional file 1: Figure S1B). Presence of Mel526 cells did not appear to inhibit DCs in this set-up, but a slight reduction of MHC class I and II molecules was observed in co-cultured DCs unless when stimulated with LOAd732 (Additional file 1: Figure S1B). Also, both LOAd703 and LOAd732-stimulated DCs secreted generally lower levels of IFNγ and higher levels of IL-10 in the presence of the Mel526 cells compared to culture alone. Hence, both LOAd703 and LOAd732 induced a reversed IL-12/IL-10 ratio in the co-culture (Additional file 1: Figure S1B), which may be either due to changes induced by the tumor cells or because the DCs are activated to an even greater degree in co-culture and therefore secrete higher IL-10 in return.

### LOAd732-activated DCs were able to expand antigen-specific T cells

**Fig. 5:**
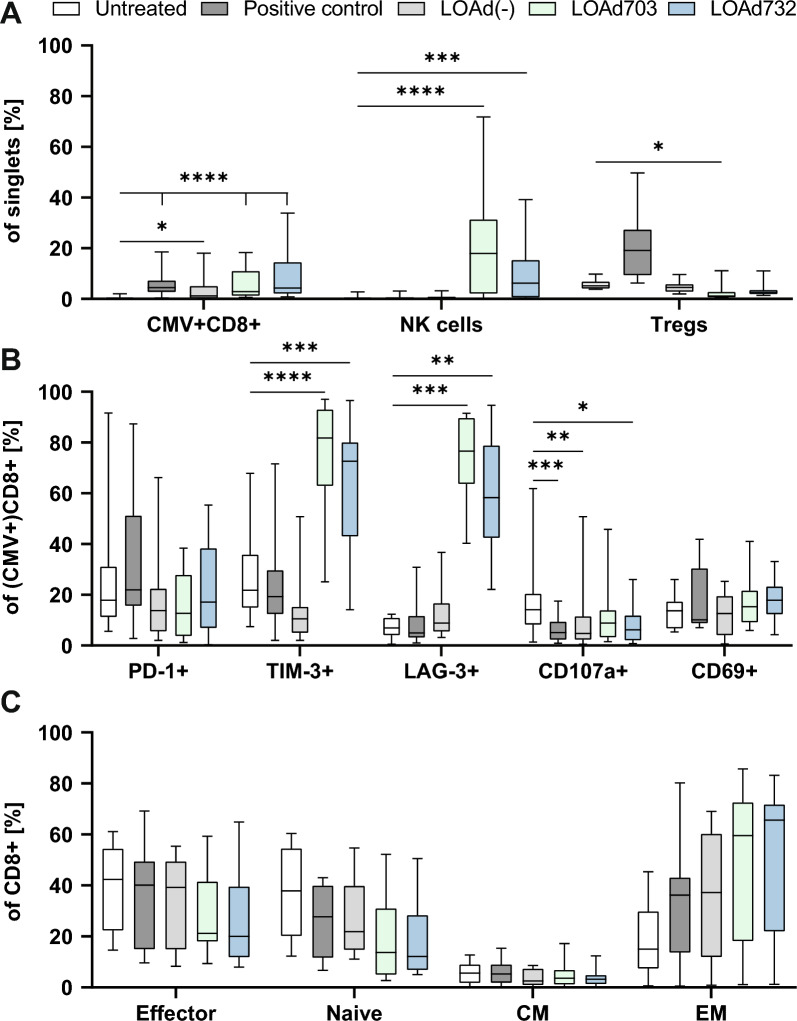
Expansion of antigen-specific T cells in a CMV model system. Monocytes and T cells were isolated from peripheral blood mononuclear cells of donors screened for having CMV-specific T cells. Monocytes were differentiated with GM-CSF/IL-4 to immature dendritic cells (DCs). DCs were infected with LOAd(-), LOAd703 or LOAd732, or stimulated with Poly(I:C)/TNFα (positive control), or left untreated. 24 h later, DCs were pulsed with the CMV peptide pp65 and co-cultured with autologous T cells. After 11 days of co-culture, cells were harvested and analyzed with flow cytometry. In A, the percentage of expanded CMV-specific T cells (CD3 + CD8 + CMVtet +), natural killer (NK) cells (CD3-CD56 + /CD16 +) and regulatory T cells (Tregs: CD3 + CD4 + CD25 + CD127low/-) is shown (**A**). In B, the percentage of exhaustion/activation markers on CMV-specific T cells is shown (**B**). Note, CD69 expression is analyzed on CD8 + T cells. The graph in C displays the different phenotypes of CD8 + T cells (Effector: CD45RA + CCR7-, Naïve: CD45RA + CCR7 + , Central memory (CM): CD45RA-CCR7 + , Effector memory (EM): CD45RA-CCR7-) (**C**). Box plots show the median and quartiles and whiskers display the minimum and maximum (n = 20; note for Tregs, LAG-3, CD69 and graphs in C n = 9). Statistical differences between treatments and untreated cells were determined with Kruskal–Wallis test followed by Dunn’s multiple comparison test (*p < 0.05, **p < 0.01, ***p < 0.001, ****p < 0.0001)

To confirm the functionality of LOAd732-activated DCs, we aimed to determine the DCs’ ability to expand and stimulate antigen-specific T cells. For this, we utilized a cytomegalovirus (CMV) model system, in which DCs were generated from blood donors positive for CMV-specific T cells. These DCs were infected with the LOAd viruses or activated with a maturation cocktail containing TNFα and Poly(I:C) as positive control, and then pulsed with a CMV peptide and co-cultured with autologous T cells. After 11 days of co-culture, the samples were analyzed for the expansion of CMV-specific T cells with flow cytometry (gating strategies are shown in Additional file 2 and Additional file 3). Both LOAd703- and LOAd732-activated DCs successfully expanded the CMV-specific T cells with slightly higher levels observed with LOAd732. Even though T cells were isolated initially, a small fraction of other cells, including NK cells remained in the T-cell fraction. Since 4-1BBL is a very potent NK cell stimulator, we also checked for the expansion of NK cells, which were indeed expanded to a large extent in LOAd703 and LOAd732 samples. Further, the expansion of regulatory T cells (Tregs) was determined since IL-2 expressed by LOAd732 may drive proliferation of Tregs. Nevertheless, Tregs were only highly increased in positive control samples and not with any of the viruses, which showed lower levels compared to both untreated and positive control samples (Fig. 5A). The CMV-specific T cells were further analyzed for their expression of exhaustion and activation molecules. PD-1 expression was highest in positive control samples, whereas both LOAd703 and LOAd732 resulted in substantial levels of TIM-3 and LAG-3. CD107a expression was highest in untreated control samples, whereas CD69 expression on CD8 + T cells was somewhat higher in LOAd703 and LOAd732 samples (Fig. 5B). Lastly, we determined the memory phenotype of the CD8 + T cells based on the expression of CD45RA and CCR7 (Effector cells: CD45RA + CCR7-, naïve cells: CD45RA + CCR7 + , central memory (CM) cells: CD45RA-CCR7 + , effector memory (EM) cells: CD45RA-CCR7-). Cells stimulated by LOAd703 and LOAd732 were largely of an effector memory (EM) phenotype (CD45RA-CCR7-). Note that two donors had only CD45RA + CD8 T cells and if these outliers are removed, the expansion of EM T cells would be significant (Fig. 5C).

### LOAd732-induced expansion of antigen-specific T cells was not impaired under suppressive conditions

**Fig. 6:**
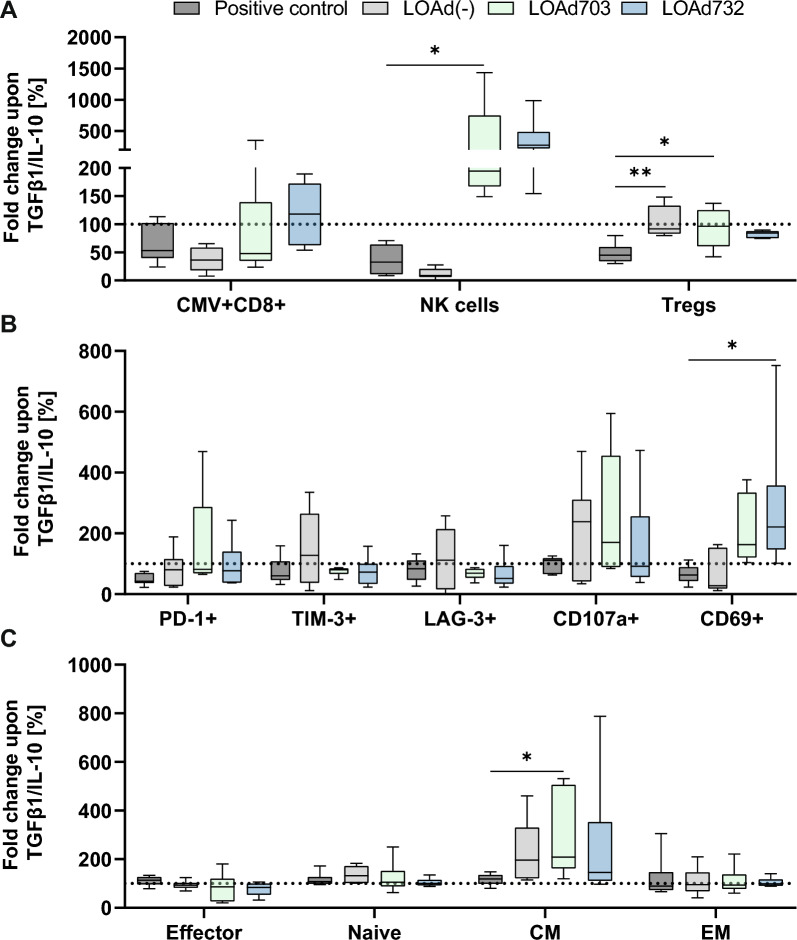
Expansion of antigen-specific T cells in a CMV model system under suppressive conditions. Monocytes and T cells were isolated from peripheral blood mononuclear cells of donors screened for having CMV-specific T cells. Monocytes were differentiated with GM-CSF/IL-4 to immature dendritic cells (DCs). DCs were infected with LOAd(-), LOAd703 or LOAd7032, or stimulated with Poly(I:C)/TNFα (positive control), or left untreated. 24 h later, DCs were pulsed with the CMV peptide pp65 and co-cultured with autologous T cells with and without the addition of TGF-β1 and IL-10. After 11 days of co-culture, cells were harvested and analyzed with flow cytometry. The box plots show the median and quartiles and whiskers display the minimum and maximum (n = 6). The data is displayed as a percentage fold change compared to control conditions. In A, the expanded cell types are shown (CMV-specific T cells (CD3 + CD8 + CMVtet +), natural killer (NK) cells (CD3-CD56 +/ CD16 +) and regulatory T cells (Tregs: CD3 + CD4 + CD25 + CD127low/-)) (**A**). In B, exhaustion/activation markers on CMV-specific T cells are shown. Note, CD69 expression is analyzed on CD8 + T cells (**B**). In C, the different phenotypes of CD8 + T cells are displayed (Effector: CD45RA + CCR7-, Naïve: CD45RA + CCR7 + , Central memory (CM): CD45RA-CCR7 + , Effector memory (EM): CD45RA-CCR7-) (**C**). Statistical differences between LOAd samples and positive control samples were determined with Kruskal–Wallis test followed by Dunn’s multiple comparison test (*p < 0.05, **p < 0.01)

Lastly, we wanted to explore the viruses’ ability to expand antigen-specific T cells under suppressive conditions that the T cells would be subjected to in the TME. TGF-β1 and IL-10 are major tumor-supporting cytokines in the TME of many cancers, including malignant melanoma, which have similar functions and are working together in a positive feedback loop to restrict immune responses. They exert their suppressive function through impairing the antigen presentation capabilities of DCs, by hindering the generation of effector T cells and by promoting Treg conversion [16, 17]. Therefore, TGF-β1 and IL-10 were added to the co-cultures in the CMV model system. The same donor was also run in parallel under standard control conditions and the data is displayed as a fold change over control. As hypothesized, the expansion of CMV-specific T cells was reduced upon the addition of suppressive cytokines in the positive control samples, but strikingly LOAd732-stimulation still resulted in a similar or even slightly higher expansion of CMV-specific T cells. NK cell proliferation was driven even further under suppressive conditions in LOAd703 and LOAd732 samples, whereas Tregs were still not further expanded with LOAd732 (Fig. 6A). The expression of PD-1, TIM-3 and LAG-3 was largely unchanged or slightly reduced compared to control conditions. Interestingly, the activation markers CD107a and CD69 were increased in presence of TGF-β1 and IL-10 in LOAd703 and LOAd732 samples (Fig. 6B). Regarding the memory phenotype, the suppressive conditions appeared to favor the expansion of central memory CD8 + T cells (CD45RA-CCR7 +) in all LOAd samples (Fig. 6C).

### LOAd732-stimulated cells retained an enhanced activation profile under suppressive conditions

**Fig. 7:**
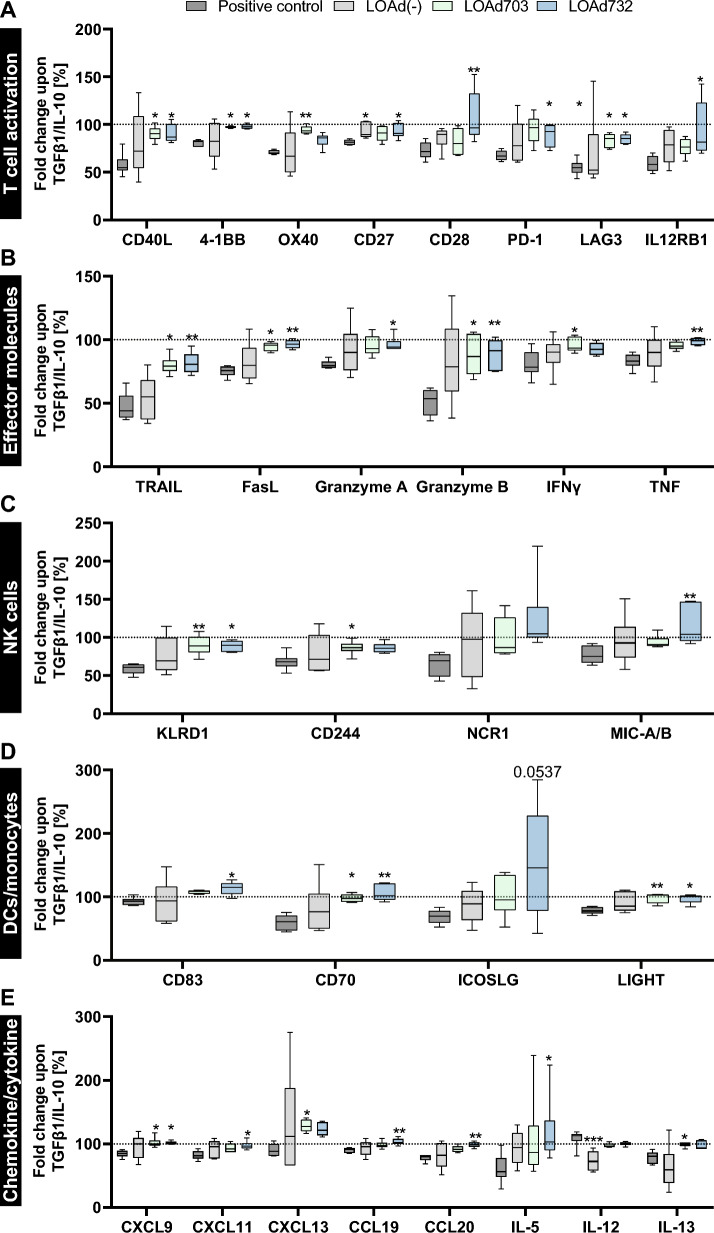
Proteomic analysis of cell culture supernatants from the CMV model under suppressive conditions. Monocytes and T cells were isolated from peripheral blood mononuclear cells of donors screened for having CMV-specific T cells. Monocytes were differentiated with GM-CSF/IL-4 to immature dendritic cells (DCs). DCs were infected with LOAd(-), LOAd703 or LOAd732, or stimulated with Poly(I:C)/TNFα (positive control), or left untreated. 24 h later, DCs were pulsed with the CMV peptide pp65 and co-cultured with autologous T cells with and without the addition of TGF-β1 and IL-10. After 11 days of co-culture, cell culture supernatants were harvested and analyzed with Olink Target 96 Immuno-Oncology multiplex assay. The box plots show the median and quartiles and whiskers display the minimum and maximum (n = 6). The data is displayed as a percentage fold change compared to control conditions. In A, markers connected to T-cell activation are shown (**A**). B displays T and natural killer (NK) cell effector molecules (**B**). Graphs in C and D show NK cell specific (**C**) and DC specific markers (**D**), respectively. E displays chemokines and cytokines (**E**). Statistical differences between LOAd samples and positive control samples were determined with Kruskal–Wallis test followed by Dunn’s multiple comparison test (*p < 0.05, **p < 0.01, ***p < 0.001)

The cell culture supernatants of the CMV model experiments under suppressive conditions were analyzed with Olink target multiplex assay. This analysis revealed that both LOAd703 and LOAd732, but in particular LOAd732, could withstand a TGF-β1/IL-10 induced reduction of T-cell activation markers, such as CD40L, 4-1BB, OX40, CD27, CD28, PD-1, LAG3 and IL12Rβ1 (Fig. 7A) and effector molecules including TRAIL, FasL, Granzyme A and B, IFNγ and TNF (Fig. 7B). Moreover, expression of molecules connected to NK cell function (Fig. 7C) and DC activation (Fig. 7D) was retained in LOAd703/LOAd732 samples under suppressive conditions. Lastly, also a multitude of chemokines and cytokines were not decreased by addition of TGF-β1/IL-10 when the cells were stimulated with either LOAd703 or LOAd732 (Fig. 7E). These included important T-cell recruiting chemokines, CXCL9 and CXCL11, and chemokines involved in DC migration (CCL19, CCL20). The absolute expression data of both control and suppressive conditions is shown in Additional file 4, Additional file 5, Additional file 6, Additional file 7 and Additional file 8.

## Discussion

In this study, we introduced a novel oncolytic adenovirus, LOAd732, expressing three immunostimulatory molecules central in the induction of antigen-specific T-cell responses. As of now, most armed oncolytic viruses evaluated in the clinic only express one therapeutic transgene and the most frequently used transgene is granulocyte–macrophage colony-stimulating factor (GM-CSF), which targets DC stimulation [18]. To our knowledge, there have only been few approaches of expressing multiple transgenes from either the same vector or from a cocktail of transgene-expressing oncolytic viruses [19–21]. Adenoviral vectors have long been utilized in gene therapy for their capacity to encode for large transgenes and the modification to the LOAd viral backbone enables the simultaneous expression of three transgenes in LOAd732; TMZ-CD40L, 4-1BBL and IL-2 [13, 22]. The expression of all three transgenes was demonstrated in LOAd732-infected melanoma cell lines and DCs and the addition of the IL-2 transgene did not impair the oncolytic function of the virus compared to LOAd703 expressing only TMZ-CD40L and 4-1BBL.

As the LOAd viruses neither can infect murine cells lacking the receptor for viral entry nor replicate in murine cells, and the transgenes have limited cross reactivity with the murine counterparts [23–25], we investigated the proposed mechanism of action of the transgenes in human in vitro models. Firstly, the expression of TMZ-CD40L and ligation of CD40 on DCs promotes DC activation and enhances their capability to present antigens and elicit T-cell responses. Secondly, the stimulation of T cells is then supported by the expression of 4-1BBL and ligation of 4-1BB on T cells. Lastly, the expression of IL-2 supports the proliferation of T cells. In addition, 4-1BBL and IL-2 signaling also leads to stimulation of NK cells [8–10]. To achieve effective T-cell responses and not to induce immune tolerance, it is pivotal to adequately mature DCs to express co-stimulatory molecules and cytokines [26]. For example, in progressing malignant melanoma patients, immature DCs are frequently increased in the tumor lesion and draining lymph nodes and are linked to primary resistance to checkpoint blockade therapy [27, 28]. Triggering of CD40 on DCs, together with the stimulation of Toll-like receptors (TLR), is recognized to lead to robust DC activation [8]. Hence, expression of CD40L by LOAd703/LOAd732 provides both signals as immune recognition of adenoviruses is thought to be mediated by TLRs, such as TLR-2 and TLR-9 [29, 30]. Direct LOAd732 infection in DCs resulted in a lower DC activation profile compared to LOAd703, but LOAd732-stimulated DCs showed a higher IL-12/IL-10 ratio, which may favor Th1 responses. As LOAd703 induced high IL-10 secretion, the DCs were possibly overactivated in this experimental setting by LOAd703 and thus produced IL-10 as a feedback response. The overall lower activation in LOAd732-stimulated DCs may be due to the lower CD40L and 4-1BBL expression post LOAd732 infection as the activation level could be restored when LOAd732-infected melanoma cells were co-cultured with DCs instead. This set-up is also better mimicking the events in the TME, as intratumoral injections of the virus will primarily lead to the infection of tumor cells. The resulting high expression of transgenes together with the oncolytic effect and subsequent release of viral particles, Damage-associated molecular patterns (DAMPs) and tumor antigens likely explain the higher activation potential of co-cultured immature DCs. LOAd703/LOAd732 upregulated not only co-stimulatory molecules required for T-cell stimulation, but also the chemokine receptor CCR7 and the adhesion molecule ICAM-1, which are crucial for lymph node homing and the initiation of a systemic response [31, 32]. Interestingly, IL-6, which has been proposed to inhibit DC maturation [33], was only minimally secreted post LOAd732 infection in DCs and LOAd732 induced high levels of CXCL10 comparable to LOAd703. Preclinical studies have demonstrated the importance of DC-derived CXCL10 in the recruitment of effector T cells to the TME [34]. In melanoma patients, CXCL10 expressed by immune cells in the tumor has been identified as a predictive factor for response to immunotherapy [35]. In cancer patients and in co-culture with tumor cells, DCs have been observed to express reduced levels of MHC molecules [36, 37]. Likewise, DCs co-cultured with Mel526 cells displayed a reduction in MHC class I and II molecules, but LOAd732-infected DCs retained the expression levels, indicating that LOAd732-activated DCs may withstand tumor-induced suppression better. This may be a direct effect of IL-2, as there have been reports of IL-2 induced upregulation of MHC class II in neutrophils and of MHC class I in papillary thyroid cancer cells [38, 39], but at the same time monocyte-derived DCs were found to neither express IL-2Rβ nor respond to IL-2 [40]. Hence, it might rather be an effect caused by changes induced in the infected tumor cells by LOAd732, which then prevent the tumor-induced MHC downregulation. All in all, both LOAd703 and LOAd732-matured DCs displayed a high activation profile, but this in turn led to high expression levels of PD-L1, which can restrict the cytokine production and proliferation of activated T cells [41]. Thus, combination therapy with PD-1/PD-L1 blockade may be warranted to establish an efficient anti-tumor immune response. In a murine melanoma model, combination of a murine version of LOAd703 with anti-PD-L1 antibodies resulted in the control of injected tumors and generation of abscopal anti-tumor immune responses [4]. The combination of LOAd703 with atezolizumab is currently being assessed in clinical trials for the treatment of pancreatic cancer and malignant melanoma (NCT02705196, NCT04123470).

Further, we confirmed the functionality of LOAd703/732-matured DCs in a CMV model system, in which antigen-specific T cells could be activated and expanded alongside with NK cells. These effects were most likely mediated by the transgenes 4-1BBL and IL-2, as both stimulate T- and NK-cell responses [9, 10]. Driving NK-cell responses may be particularly advantageous in tumors that evade a T-cell response by downregulating MHC class I molecules, which is a common resistance mechanism to checkpoint blockade therapy in malignant melanoma [42, 43]. As LOAd732 additionally expresses IL-2, we expected an enhanced expansion with LOAd732-infected DCs. This was especially evident upon the addition of the suppressive factors TGF-β1 and IL-10, as T-cell responses were inhibited in the control condition, but not with LOAd732-induced stimulation. The higher IL-12/IL-10 ratio seen in LOAd732-infected DCs may make the DCs less sensitive to the suppressive factors compared to LOAd703-infected DCs that may be overstimulated already and thus LOAd732 may stimulate T-cell responses better under suppressive conditions. While IL-2 stimulates effector T cells, it can also drive the expansion of Tregs. Favorably, we did not observe increased levels of Tregs with LOAd732, not in control nor under suppressive conditions. Probably, the overall pro-inflammatory signals induced by LOAd732 favor an effector T-cell phenotype over a Treg phenotype [44]. When further investigating the CD8 + T-cell phenotype, we noticed that LOAd732 shifted the T cells toward an effector memory phenotype. This phenotype may be favorable for an anti-tumor immune response, as effector memory cells were found to be expanded in tumors and in blood of melanoma patients responding to PD-1 blockade [45, 46]. LOAd703/732-expanded antigen-specific T cells highly expressed the checkpoint molecules TIM-3 and LAG-3, and to a lesser extent PD-1. Hence, it would be of interest to also study the combination of checkpoint inhibitors targeting TIM-3 or LAG-3 in combination with LOAd732. In vivo studies with a murine version of LOAd703 in combination with anti-TIM-3 have shown enhanced tumor control in the otherwise checkpoint-resistant B16 melanoma model [4].

Proteomic analysis of the CMV model system with TGF-β1/IL-10 further confirmed the immunosuppressive effects of these cytokines in the positive control samples, as this led to a downregulation of a multitude of markers involved in T-cell, NK-cell and DC activation as well as effector molecules, chemokines and cytokines. TGF-β1 and IL-10 are central cytokines with pleiotropic effects in regulating and controlling T-cell responses. IL-10 acts on antigen-presenting cells to inhibit their potential to stimulate T cells by hampering the expression of pro-inflammatory cytokines, MHC and co-stimulatory molecules, but IL-10 can also directly affect T cells by inhibiting the CD28 co-stimulatory pathway [47, 48]. TGF-β1 mainly inhibits the proliferation, activation and effector functions of T cells and regulates T-cell differentiation [49]. Strikingly, especially LOAd732 stimulation could counteract TGF-β1/IL-10-induced suppression and maintained expression levels of the investigated molecules. Some analytes appeared even slightly increased with LOAd732 in suppressive conditions, which included CD28, IL-12Rβ1, NCR1, MIC-A/B, CD83, CD70, ICOS ligand, CXCL9, CXCL13, CCL19 and IL-5. This exemplifies the widespread immunostimulatory effects of LOAd732 therapy, since it enhanced expression of important co-stimulatory molecules involved in the immunological synapse of DCs and T cells (CD28, CD70, ICOS ligand) [50], as well as leading to enhanced maturation of DCs (CD83) [51] and triggering of NK cells (NCR1, MIC-A/B) [52]. Moreover, factors crucial for the activation and recruitment of T cells and DCs (IL-12Rβ1, CXCL9, CCL19) [53, 54] and the stimulation of B cells and Th2 responses (CXCL13, IL-5) [55, 56] were increased with LOAd732. It remains to be investigated if this effect is directly related to the expression of IL-2, or if the LOAd732 infection in DCs leads to less overactivated DCs that are more resistant to suppressive conditions and maintain their T-cell stimulatory function. For example, others have found that IL-2 can induce CD70 and IL-5 expression in T cells, as well as IL-12Rβ1 expression on NK cells [57–59]. Also, the suppressive effect of TGF-β1 on T-cell proliferation was found to be mediated through the inhibition of IL-2 production [60]. Therefore, the additional expression of IL-2 provided by LOAd732 was probably counteracting this effect.

Historically, IL-2 became the first successful immunotherapy due to its potent T-cell stimulatory properties [61]. However, systemic treatment is problematic due to a short half-life and severe toxicity. In addition, IL-2 induced Treg expansion may hamper the immunostimulatory effects [62]. These hurdles of IL-2 therapy can potentially be overcome by inducing IL-2 expression locally in the TME by an oncolytic virus, such as LOAd732. Due to LOAd732’s seemingly better effect in immunosuppressive conditions, it would be of interest to further study the mechanistic effects in immune cells isolated from malignant melanoma patients, in human ex vivo tumor models and to explore the combination with different checkpoint inhibitors.

## Conclusions

LOAd732 is a novel immunostimulatory gene therapy based on an oncolytic adenovirus that expresses three transgenes, which are essential for mediating anti-tumor immune responses. Arming the oncolytic virus with three transgenes was feasible and the immunostimulatory capacity of LOAd732 was demonstrated by the activation of DCs and subsequent generation of antigen-specific T-cell responses alongside with NK cell stimulation. Strikingly, these effects were maintained even under imunosuppressive conditions commonly present in the TME. Hence, LOAd732 therapy represents an interesting new approach for the treatment of cancers.

## Supplementary Information

**Additional file 1: Figure S1.** Activation of dendritic cells by LOAd viruses. Monocytes were isolated from peripheral blood mononuclear cells and cultured with GM-CSF/IL-4 to induce immature dendritic cells (DCs). DCs were infected with LOAd(-), LOAd703 or LOAd732, or left untreated. 48 h post infection, cell culture supernatants and cells were harvested and analyzed for DC maturation with flow cytometry and multiplex analysis. Flow cytometry results in A are displayed as relative mean fluorescence intensity (RMFI) compared to matched isotype control antibodies and cytokine levels in culture supernatants are shown in pg/mL. In B, uninfected DCs were co-cultured with infected or untreated Mel526 cells and the percentage fold change compared to DCs cultured and infected alone was calculated. Bar graphs display the mean ± SD (n = 7 for A and n = 3 for B). Statistical differences between LOAd-infected and untreated cells were determined with Kruskal–Wallis test followed by Dunn’s multiple comparison test (**p < 0.01, ***p < 0.001, ****p < 0.0001).

**Additional file 2: Figure S2.** Flow cytometry gating strategy CMV-specific T cells. Cells were first gated on FSC-A vs SSC-A and then further gated for viable cells based on Zombie NIR staining. Doublets were excluded by gating FSC-A vs FSC-H. Singlets were gated for CD3 + cells and these were further gated for CD8 expression and positive CMV tetramer staining. CD3 + CD8 + CMVtet + cells were then analyzed for their expression of PD-1, LAG-3, TIM-3 and CD107a.

**Additional file 3: Figure S3.** Flow cytometry gating strategy natural killer cells, regulatory T cells and memory phenotype. Cells were first gated on FSC-A vs SSC-A and then further gated for singlets by gating FSC-A vs FSC-H. Singlets were gated for CD3 + cells and CD3- cells. CD3- cells were gated for CD16/CD56 expression (natural killer cells). CD3 + cells were gated for CD8 vs CD4 expression. CD4 + T cells (Q3) were analyzed for regulatory T cells by gating CD127-CD25 + cells. CD8 + T cells (Q1) were analyzed for CD69 expression and for their memory phenotype by gating CD45RA vs CCR7 (Q5: CD45RA + CCR7- effector cells, Q6: CD45RA + CCR7 + naive cells, Q7: CD45RACCR7 + central memory cells, Q8: CD45RA-CCR7- effector memory cells).

**Additional file 4: Figure S4.** Proteomic analysis of cell culture supernatants of the CMV model: T cell activation. Monocytes and T cells were isolated from peripheral blood mononuclear cells of donors screened for having CMV-specific T cells. Monocytes were differentiated with GM-CSF/IL-4 to immature dendritic cells (DCs). DCs were infected with LOAd(-), LOAd703 or LOAd732, or stimulated with Poly(I:C)/TNFα (positive control), or left untreated. 24 h later, DCs were pulsed with the CMV peptide pp65 and co-cultured with autologous T cells with and without the addition of TGF-β1 and IL-10. After 11 days of co-culture, cell culture supernatants were harvested and analyzed with Olink Target 96 Immuno-Oncology multiplex assay. The bar graphs show the mean ± SD (n = 6). Black bars: positive control, gray bars: LOAd(-), green bars: LOAd703, blue bars: LOAd732.

**Additional file 5: Figure S5.** Proteomic analysis of cell culture supernatants of the CMV model: Effector molecules. Monocytes and T cells were isolated from peripheral blood mononuclear cells of donors screened for having CMV-specific T cells. Monocytes were differentiated with GM-CSF/IL-4 to immature dendritic cells (DCs). DCs were infected with LOAd(-), LOAd703 or LOAd732, or stimulated with Poly(I:C)/TNFα (positive control), or left untreated. 24 h later, DCs were pulsed with the CMV peptide pp65 and co-cultured with autologous T cells with and without the addition of TGF-β1 and IL-10. After 11 days of co-culture, cell culture supernatants were harvested and analyzed with Olink Target 96 Immuno-Oncology multiplex assay. The bar graphs show the mean ± SD (n = 6). Black bars: positive control, gray bars: LOAd(-), green bars: LOAd703, blue bars: LOAd732.

**Additional file 6: Figure S6.** Proteomic analysis of cell culture supernatants of the CMV model: NK cells. Monocytes and T cells were isolated from peripheral blood mononuclear cells of donors screened for having CMV-specific T cells. Monocytes were differentiated with GM-CSF/IL-4 to immature dendritic cells (DCs). DCs were infected with LOAd(-), LOAd703 or LOAd732, or stimulated with Poly(I:C)/TNFα (positive control), or left untreated. 24 h later, DCs were pulsed with the CMV peptide pp65 and co-cultured with autologous T cells with and without the addition of TGF-β1 and IL-10. After 11 days of co-culture, cell culture supernatants were harvested and analyzed with Olink Target 96 Immuno-Oncology multiplex assay. The bar graphs show the mean ± SD (n = 6). Black bars: positive control, gray bars: LOAd(-), green bars: LOAd703, blue bars: LOAd732.

**Additional file 7: Figure S7.** Proteomic analysis of cell culture supernatants of the CMV model: DCs/monocytes. Monocytes and T cells were isolated from peripheral blood mononuclear cells of donors screened for having CMV-specific T cells. Monocytes were differentiated with GM-CSF/IL-4 to immature dendritic cells (DCs). DCs were infected with LOAd(-), LOAd703 or LOAd732, or stimulated with Poly(I:C)/TNFα (positive control), or left untreated. 24 h later, DCs were pulsed with the CMV peptide pp65 and co-cultured with autologous T cells with and without the addition of TGF-β1 and IL-10. After 11 days of co-culture, cell culture supernatants were harvested and analyzed with Olink Target 96 Immuno-Oncology multiplex assay. The bar graphs show the mean ± SD (n = 6). Black bars: positive control, gray bars: LOAd(-), green bars: LOAd703, blue bars: LOAd732.

**Additional file 8: Figure S8.** Proteomic analysis of cell culture supernatants of the CMV model: Chemokines/cytokines. Monocytes and T cells were isolated from peripheral blood mononuclear cells of donors screened for having CMV-specific T cells. Monocytes were differentiated with GM-CSF/IL-4 to immature dendritic cells (DCs). DCs were infected with LOAd(-), LOAd703 or LOAd732, or stimulated with Poly(I:C)/TNFα (positive control), or left untreated. 24 h later, DCs were pulsed with the CMV peptide pp65 and co-cultured with autologous T cells with and without the addition of TGF-β1 and IL-10. After 11 days of co-culture, cell culture supernatants were harvested and analyzed with Olink Target 96 Immuno-Oncology multiplex assay. The bar graphs show the mean ± SD (n = 6). Black bars: positive control, gray bars: LOAd(-), green bars: LOAd703, blue bars: LOAd732.

## Data Availability

The datasets used and/or analyzed during the current study are available from the corresponding author on reasonable request.

## Abbreviations

CMV: Cytomegalovirus
DC: Dendritic cell
EM: Effector memory
FBS: Fetal bovine serum
FFU: Fluorescent forming units
LOAd: Lokon oncolytic adenovirus
NK: Natural killer
PBMCs: Peripheral blood mononuclear cells
PeSt: Penicillin/streptomycin
TLR: Toll-like receptor
TME: Tumor microenvironment
TMZ-CD40L: Trimerized membrane-bound CD40L
Tregs: Regulatory T cells
