# Supplementary material for: Immunostimulatory gene therapy targeting CD40, 4-1BB and IL-2R activates DCs and stimulates antigen-specific T-cell and NK-cell responses in melanoma models
Source: J Transl Med. 2023 Jul 27;21:506. doi: 10.1186/s12967-023-04374-2 (PMC10373363; doi:10.1186/s12967-023-04374-2)
Supplement: Supplementary file 1 — Additional file 1: Figure S1. Activation of dendritic cells by LOAd viruses. Monocytes were isolated from peripheral blood mononuclear cells and cultured with GM-CSF/IL-4 to induce immature dendritic cells (DCs). DCs were infected with LOAd(-), LOAd703 or LOAd732, or left untreated. 48 h post infection, cell culture supernatants and cells were harvested and analyzed for DC maturation with flow cytometry and multiplex analysis. Flow cytometry results in A are displayed as relative mean fluorescence intensity (RMFI) compared to matched isotype control antibodies and cytokine levels in culture supernatants are shown in pg/mL. In B, uninfected DCs were co-cultured with infected or untreated Mel526 cells and the percentage fold change compared to DCs cultured and infected alone was calculated. Bar graphs display the mean ± SD (n = 7 for A and n = 3 for B). Statistical differences between LOAd-infected and untreated cells were determined with Kruskal–Wallis test followed by Dunn’s multiple comparison test (**p < 0.01, ***p < 0.001, ****p < 0.0001). [file 12967_2023_4374_MOESM1_ESM.pdf]

Additional File 1: Figure S1

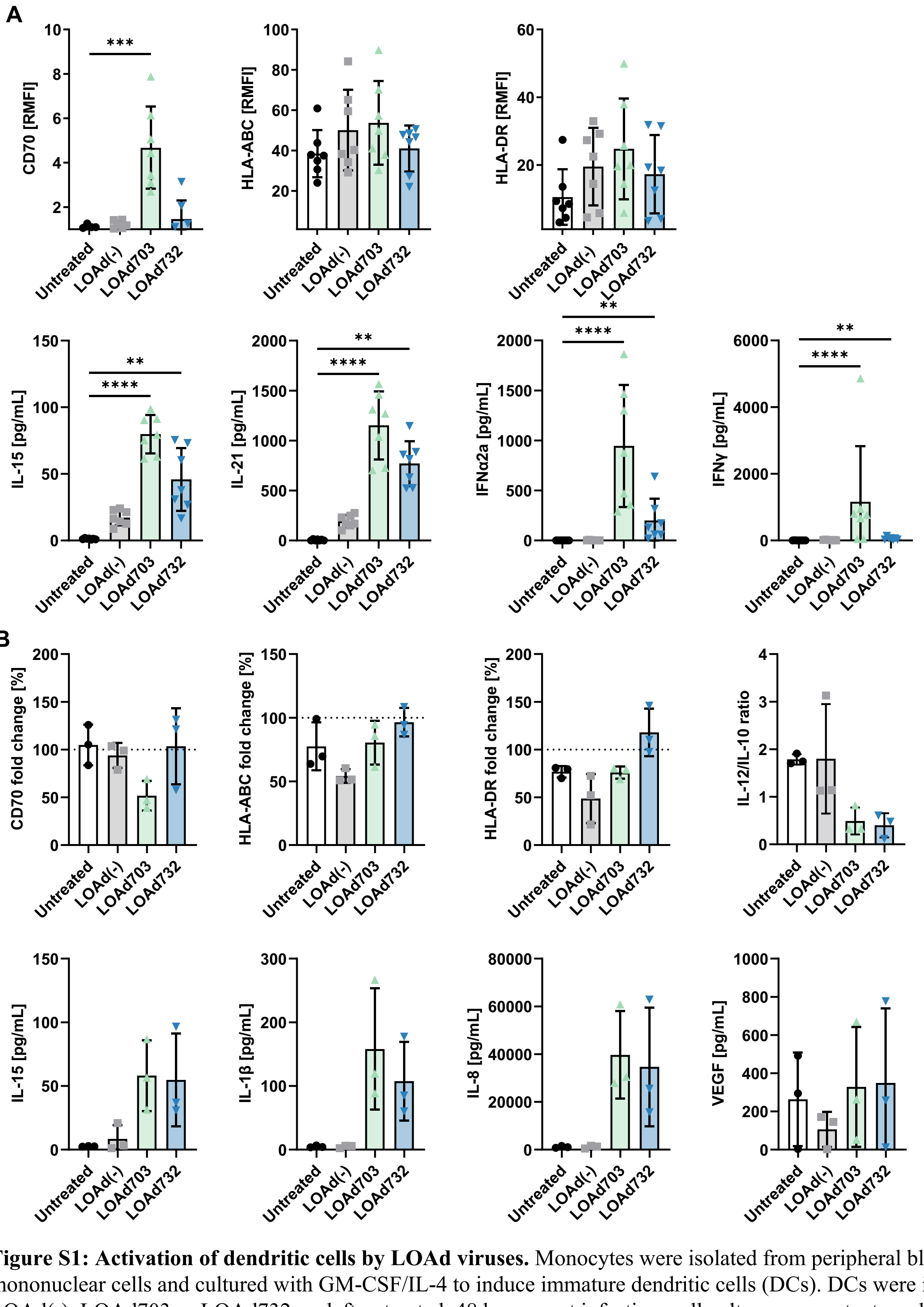

Figure S1: Activation of dendritic cells by LOAd viruses. Monocytes were isolated from peripheral blood mononuclear cells and cultured with GM-CSF/IL-4 to induce immature dendritic cells (DCs). DCs were infected with LOAd(-), LOAd703 or LOAd732, or left untreated. 48 hours post infection, cell culture supernatants and cells were harvested and analyzed for DC maturation with flow cytometry and multiplex analysis. Flow cytometry results in A are displayed as relative mean fluorescence intensity (RMFI) compared to matched isotype control antibodies and cytokine levels in culture supernatants are shown in pg/mL. In B, uninfected DCs were co-cultured with infected or untreated Mel526 cells and the percentage fold change compared to DCs cultured and infected alone was calculated. Bar graphs display the mean ± SD (n=7 for A and n=3 for B). Statistical differences between LOAd-infected and untreated cells were determined with Kruskal-Wallis test followed by Dunn's multiple comparison test (**$p<0.01$, ***$p<0.001$, ****$p<0.0001$).
