# Supplementary material for: Immunostimulatory gene therapy targeting CD40, 4-1BB and IL-2R activates DCs and stimulates antigen-specific T-cell and NK-cell responses in melanoma models
Source: J Transl Med. 2023 Jul 27;21:506. doi: 10.1186/s12967-023-04374-2 (PMC10373363; doi:10.1186/s12967-023-04374-2)
Supplement: Supplementary file 2 — Additional file 2: Figure S2. Flow cytometry gating strategy CMV-specific T cells. Cells were first gated on FSC-A vs SSC-A and then further gated for viable cells based on Zombie NIR staining. Doublets were excluded by gating FSC-A vs FSC-H. Singlets were gated for CD3 + cells and these were further gated for CD8 expression and positive CMV tetramer staining. CD3 + CD8 + CMVtet + cells were then analyzed for their expression of PD-1, LAG-3, TIM-3 and CD107a. [file 12967_2023_4374_MOESM2_ESM.pdf]

Additional File 2: Figure S2

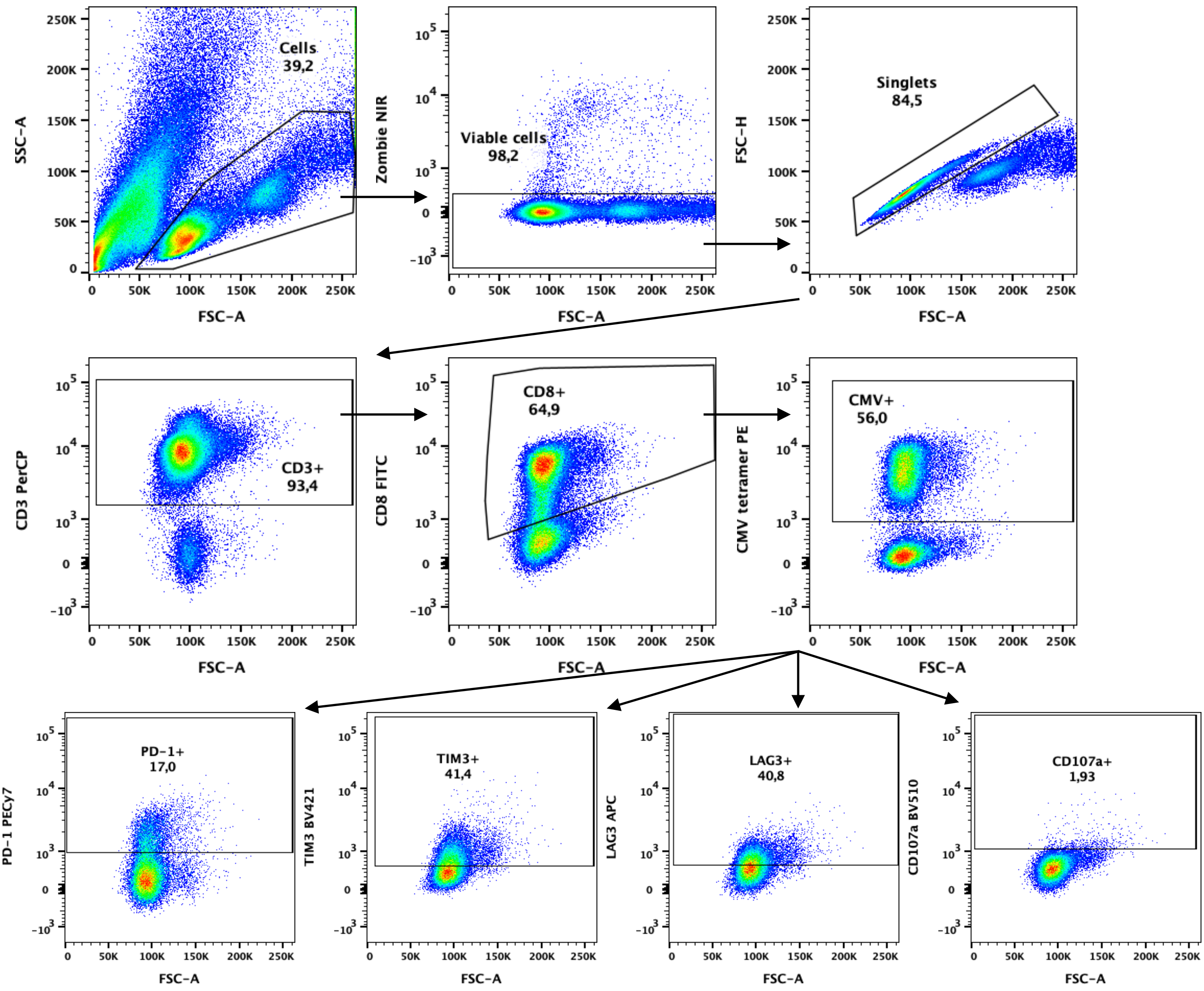

Figure S2: Flow cytometry gating strategy CMV-specific T cells. Cells were first gated on FSC-A vs SSC-A and then further gated for viable cells based on Zombie NIR staining. Doublets were excluded by gating FSC-A vs FSC-H. Singlets were gated for CD3+ cells and these were further gated for CD8 expression and positive CMV tetramer staining. CD3+CD8+CMVtet+ cells were then analyzed for their expression of PD-1, LAG-3, TIM-3 and CD107a.
