# Supplementary material for: Immunostimulatory gene therapy targeting CD40, 4-1BB and IL-2R activates DCs and stimulates antigen-specific T-cell and NK-cell responses in melanoma models
Source: J Transl Med. 2023 Jul 27;21:506. doi: 10.1186/s12967-023-04374-2 (PMC10373363; doi:10.1186/s12967-023-04374-2)
Supplement: Supplementary file 3 — Additional file 3: Figure S3. Flow cytometry gating strategy natural killer cells, regulatory T cells and memory phenotype. Cells were first gated on FSC-A vs SSC-A and then further gated for singlets by gating FSC-A vs FSC-H. Singlets were gated for CD3 + cells and CD3- cells. CD3- cells were gated for CD16/CD56 expression (natural killer cells). CD3 + cells were gated for CD8 vs CD4 expression. CD4 + T cells (Q3) were analyzed for regulatory T cells by gating CD127-CD25 + cells. CD8 + T cells (Q1) were analyzed for CD69 expression and for their memory phenotype by gating CD45RA vs CCR7 (Q5: CD45RA + CCR7- effector cells, Q6: CD45RA + CCR7 + naive cells, Q7: CD45RACCR7 + central memory cells, Q8: CD45RA-CCR7- effector memory cells). [file 12967_2023_4374_MOESM3_ESM.pdf]

Additional File 3: Figure S3

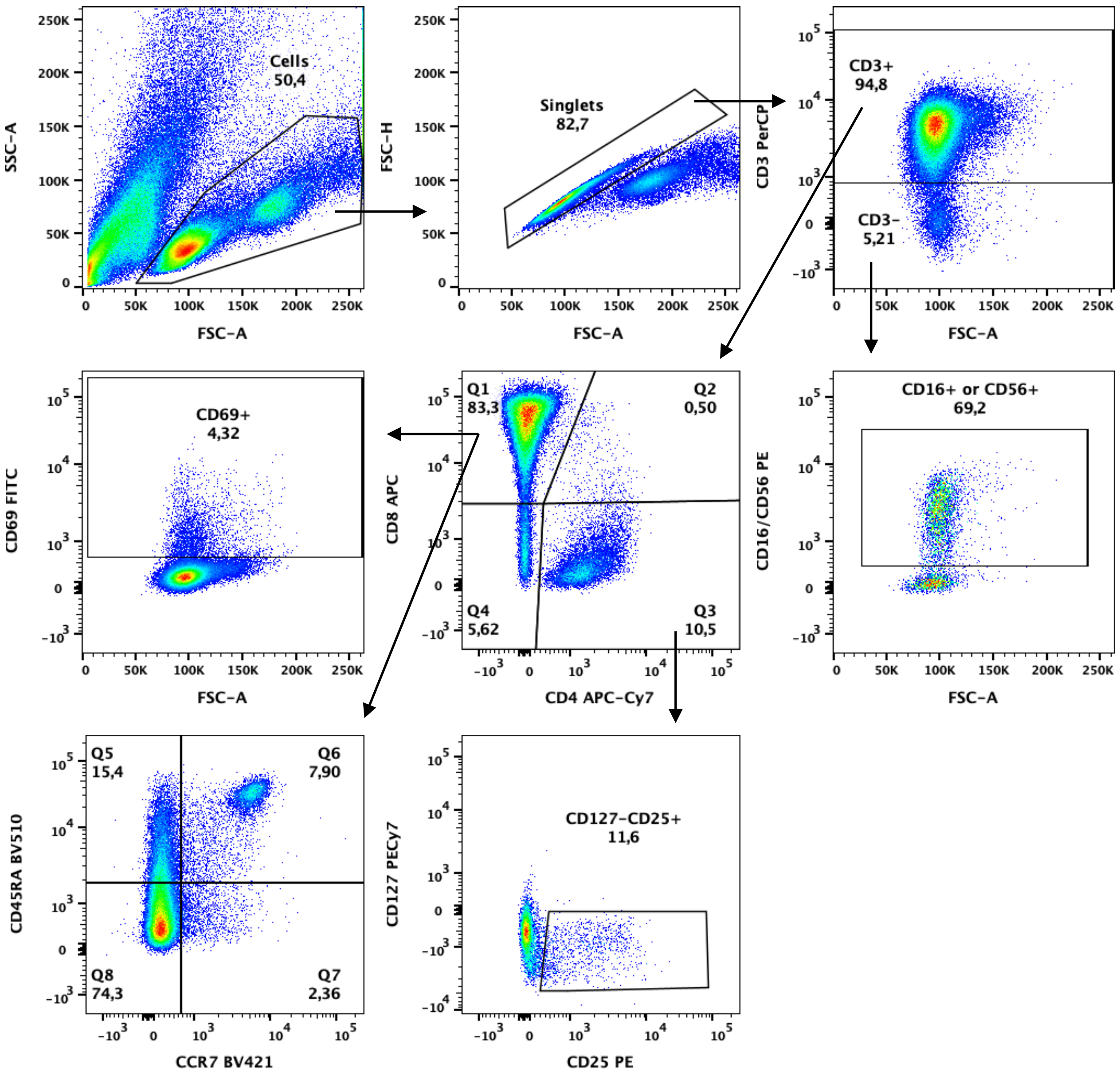

Figure S3: Flow cytometry gating strategy natural killer cells, regulatory T cells and memory phenotype. Cells were first gated on FSC-A vs SSC-A and then further gated for singlets by gating FSC-A vs FSC-H. Singlets were gated for CD3+ cells and CD3- cells. CD3- cells were gated for CD16/CD56 expression (natural killer cells). CD3+ cells were gated for CD8 vs CD4 expression. CD4+ T cells (Q3) were analyzed for regulatory T cells by gating CD127-CD25+ cells. CD8+ T cells (Q1) were analyzed for CD69 expression and for their memory phenotype by gating CD45RA vs CCR7 (Q5: CD45RA+CCR7- effector cells, Q6: CD45RA+CCR7+ naïve cells, Q7: CD45RA-CCR7+ central memory cells, Q8: CD45RA-CCR7- effector memory cells).
