# Supplementary material for: Immunostimulatory gene therapy targeting CD40, 4-1BB and IL-2R activates DCs and stimulates antigen-specific T-cell and NK-cell responses in melanoma models
Source: J Transl Med. 2023 Jul 27;21:506. doi: 10.1186/s12967-023-04374-2 (PMC10373363; doi:10.1186/s12967-023-04374-2)
Supplement: Supplementary file 7 — Additional file 7: Figure S7. Proteomic analysis of cell culture supernatants of the CMV model: DCs/monocytes. Monocytes and T cells were isolated from peripheral blood mononuclear cells of donors screened for having CMV-specific T cells. Monocytes were differentiated with GM-CSF/IL-4 to immature dendritic cells (DCs). DCs were infected with LOAd(-), LOAd703 or LOAd732, or stimulated with Poly(I:C)/TNFα (positive control), or left untreated. 24 h later, DCs were pulsed with the CMV peptide pp65 and co-cultured with autologous T cells with and without the addition of TGF-β1 and IL-10. After 11 days of co-culture, cell culture supernatants were harvested and analyzed with Olink Target 96 Immuno-Oncology multiplex assay. The bar graphs show the mean ± SD (n = 6). Black bars: positive control, gray bars: LOAd(-), green bars: LOAd703, blue bars: LOAd732. [file 12967_2023_4374_MOESM7_ESM.pdf]

Additional File 7: Figure S7

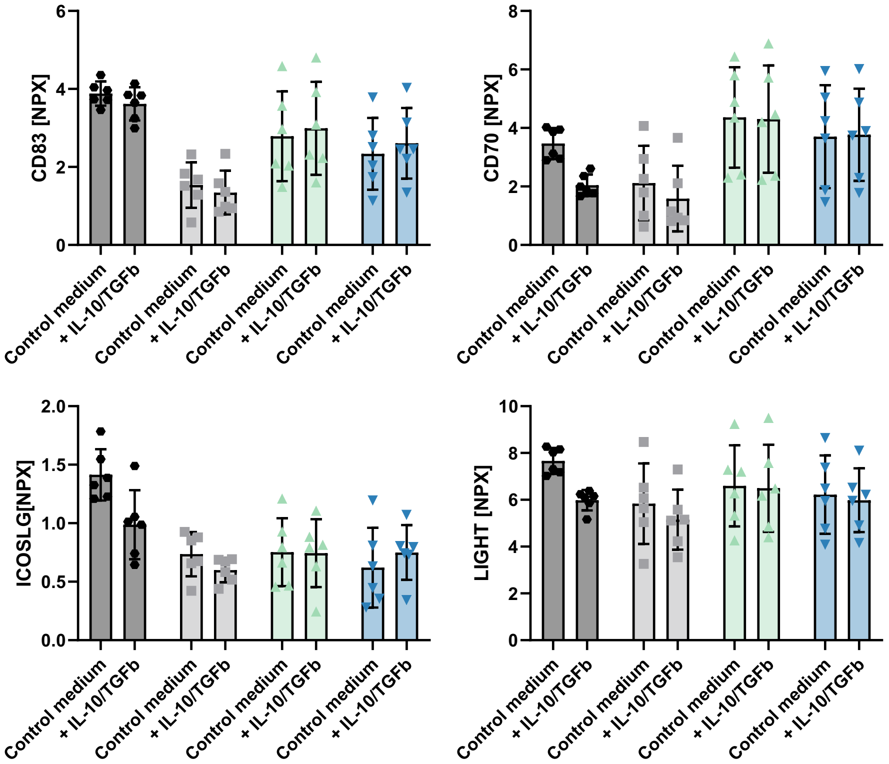

Figure S7: Proteomic analysis of cell culture supernatants of the CMV model: DCs/monocytes. Monocytes and T cells were isolated from peripheral blood mononuclear cells of donors screened for having CMV-specific T cells. Monocytes were differentiated with GM-CSF/IL-4 to immature dendritic cells (DCs). DCs were infected with LOAd(-), LOAd703 or LOAd703, or stimulated with Poly(I:C)/TNFα (positive control), or left untreated. 24 hours later, DCs were pulsed with the CMV peptide pp65 and co-cultured with autologous T cells with and without the addition of TGF-β1 and IL-10. After 11 days of co-culture, cell culture supernatants were harvested and analyzed with Olink Target 96 Immuno-Oncology multiplex assay. The bar graphs show the mean ± SD (n=6). Black bars: positive control, gray bars: LOAd(-), green bars: LOAd703, blue bars: LOAd732.
